# Impact of Adjunctive Myo-Inositol and Magnesium Therapy on Paediatric Overactive Bladder: A Retrospective Analysis

**DOI:** 10.3390/children13050604

**Published:** 2026-04-27

**Authors:** Alessandro Colletti, Michele Favro, Luciano Sangiorgio

**Affiliations:** 1 Department of Pharmaceutical Science and Technology, University of Turin, 10125 Turin, Italy; 2 Division of Urology, Maggiore della Carità Hospital, University of Eastern Piedmont, 28100 Novara, Italy; michele.favro@maggioreosp.novara.it; 3 Unit of Pediatric Surgery, Azienda USL della Valle d’Aosta, Specialist Outpatient Clinic, Via Guido Rey 3, 11100 Aosta, Italy; lsangiorgio@ausl.vda.it

**Keywords:** overactive bladder, myo-inositol, magnesium, insulin resistance

## Abstract

Background: Overactive bladder (OAB) is a common functional disorder in paediatric populations and is associated with significant psychological burden and impaired quality of life. Although oxybutynin is widely used as first-line pharmacological therapy, a substantial proportion of children exhibit incomplete symptom control or limited tolerability. Emerging evidence suggests that targeting metabolic dysfunction, oxidative stress, and neuromuscular excitability may provide additional therapeutic benefit. This retrospective observational study evaluated the clinical impact of an adjunctive nutraceutical formulation containing myo-inositol, microlipodispersed magnesium, folic acid, and vitamin C (LEVIGON™ PRO, Sanitpharma; Milan, Italy) in children with OAB receiving oxybutynin. Methods: Medical records of children diagnosed with OAB were retrospectively reviewed. After applying inclusion and exclusion criteria, 120 patients aged 5–15 years were included and allocated to two groups based on documented treatment: oxybutynin plus LEVIGON™ PRO (Group A, n = 60) or oxybutynin alone (Group B, n = 60). The primary outcome was complete daytime urinary continence at Day 112. Secondary outcomes included weekly incontinence episodes, voiding frequency, bladder wall thickness, uroflowmetry parameters, and Patient Perception of Bladder Condition (PPBC) scores. An exploratory subgroup analysis was performed in 34 children with impaired fasting glucose (ifg), assessing fasting glucose, insulin, and homa-ir. results: by day 112, complete daytime continence was achieved in 61.7% of patients in group a and 48.3% in group b (absolute risk difference 13.4%; nnt ≈ 7.5; *p* = 0.14). across secondary endpoints, the combination therapy group showed significantly greater longitudinal improvements (group × time interaction, *p* < 0.05), including reductions in weekly incontinence episodes, voiding frequency, post-void residual volume, and ppbc scores, as well as increases in mean voided volume, qmax, and reductions in bladder wall thickness. in the ifg subgroup, greater reductions in fasting glucose, fasting insulin, and homa-ir were observed in group a compared with group b (*p* < 0.01). Both treatments were well tolerated, with no serious adverse events reported. conclusions: adjunctive nutraceutical therapy combined with oxybutynin was associated with greater improvements in several clinically relevant secondary outcomes in children with OAB, with a favourable tolerability profile. Although the primary endpoint did not reach statistical significance, the overall pattern of findings may suggest a possible additive benefit; however, these findings may be influenced by residual confounding inherent to the retrospective observational design. Therefore, the results should be considered hypothesis generating and require confirmation in prospective randomized controlled trials.

## 1. Introduction

Overactive bladder (OAB) is a prevalent and distressing condition in paediatric populations, characterized by urinary urgency, often accompanied by increased daytime frequency and non-monosymptomatic nocturnal enuresis [1]. Epidemiological data indicate that OAB affects approximately 5% to 12% of children aged 5 to 10 years [2], constituting the most frequent causes of paediatric urology consultation [3,4]. OAB is associated with significant quality-of-life impairments, including low self-esteem, behavioural disturbances, and social withdrawal [5]. Conservative measures such as urotherapy and constipation management form the first-line approach [6]. However, pharmacologic interventions, primarily antimuscarinic agents such as oxybutynin, solifenacine and fesoterodine, are frequently required in refractory cases [7]. Despite proven clinical efficacy, the use of antimuscarinic agents in pediatric populations is frequently constrained by treatment-emergent adverse reactions, including xerostomia, constipation, headache, dizziness, and behavioral or attention change, as well as potential central nervous system effects [8]. The FOXY randomized controlled trial demonstrated comparable efficacy of extended-release oxybutynin and fesoterodine, but adverse effects, particularly heart rate elevation and gastrointestinal discomfort, were notable, limiting long-term adherence [7]. These limitations have prompted growing interest in complementary therapeutic strategies, particularly for patients who are refractory to standard pharmacological treatments or who experience poor adherence due to adverse effects. Approximately 20–25% of children with OAB are considered therapy-resistant to commonly used treatment options, further underscoring the need for adjunctive or alternative therapeutic strategies [9]. Paediatric OAB is likely multifactorial, with contributions from altered bladder control, behavioural factors, and comorbid functional disturbances [10]. Furthermore, comorbid neuropsychiatric conditions, especially anxiety and attention-deficit/hyperactivity disorder (ADHD), have been consistently associated with increased OAB symptom severity and therapeutic resistance [11]. An additional component is the bladder–gut–brain axis: chronic constipation, commonly coexisting with OAB, alters afferent sensory integration from the bladder via shared pelvic innervation, leading to misinterpretation of bladder fullness and urgency [12]. Rectal distension and colonic dysfunction can thus exacerbate LUTS through cross-sensitization pathways [13]. Beyond neurodevelopmental factors, emerging data suggest a strong correlation between metabolic dysfunction, particularly insulin resistance (IR), obesity, and low-grade inflammation, and the occurrence of lower urinary tract symptoms (LUTS) including OAB [14]. Mechanistic hypotheses implicate sympathetic nervous system overactivity, oxidative stress, and endothelial dysfunction as mediators linking metabolic abnormalities and bladder overactivity [15,16]. While metabolic dysfunction and IR have been associated with lower urinary tract symptoms in adults, this relationship remains insufficiently defined in paediatric OAB, and any extrapolation to children should be considered preliminary. Given that some children with OAB show suboptimal response to antimuscarinic therapy and that tolerability may limit long-term use, there is growing interest in adjunctive or alternative approaches [17]. Adjunctive nutraceutical strategies targeting selected metabolic or neuromuscular pathways may represent a potential complementary approach in children with persistent symptoms, although the supporting evidence in paediatric OAB remains preliminary. In this regard, myo-inositol, an insulin-sensitizing compound, has demonstrated efficacy in improving IR through modulation of inositol phosphoglycan secondary messengers and is widely studied in metabolic syndromes such as polycystic ovary syndrome (PCOS) [18,19]. In addition, magnesium is known to enhance inositol uptake by increasing transporter affinity and supports glycaemic regulation and neuromuscular balance [20,21]. Given this context, the present retrospective study aimed to evaluate the clinical outcomes in paediatric patients diagnosed with OAB, comparing standard antimuscarinic therapy alone to a combination treatment including adjunctive myo-inositol and micro-lipodispersed magnesium.

## 2. Materials and Methods

### 2.1. Study Design and Participants

This retrospective observational study was conducted at Ospedale Infantile Cesare Arrigo (Alessandria, Italy), by reviewing the medical records of one hundred and twenty paediatric patients aged 5 to 15 years who were diagnosed with overactive bladder (OAB) between February 2022 and May 2024.

**Figure 1:**
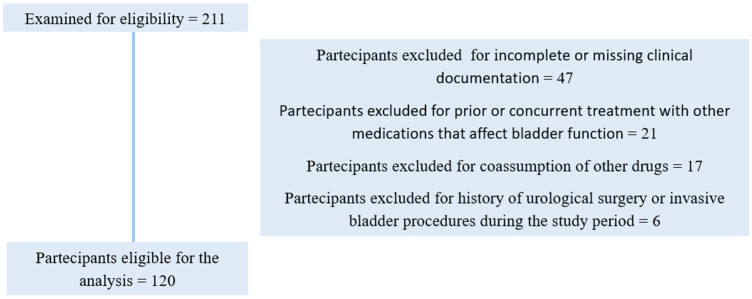
Flowchart of participant screening, exclusion criteria, and final inclusion for analysis.

The diagnosis of OAB was established in accordance with the International Children’s Continence Society (ICCS) criteria, based on clinical history, urinary urgency with or without frequency and enuresis, and, where available, bladder diary or symptom score data [4]. Patients were retrospective analysed (Figure 1) according to treatment received: standard antimuscarinic therapy (oxybutynin) alone, or the same therapy combined with adjunctive myo-inositol and micro-lipodispersed magnesium. Because of the retrospective design, treatment allocation was not protocol-driven or randomized, but reflected routine clinical prescribing as documented in the medical records. Bowel dysfunction and constipation management were not systematically recorded in a standardized format across all charts and therefore could not be formally compared between groups.

Inclusion criteria were as follows: age between 5 and 15 years; successful achievement of daytime toilet training; normal renal function; absence of complex congenital urinary tract malformations (e.g., posterior urethral valves, duplex system, ectopic ureter); no documented neurogenic bladder or spinal cord anomalies; no diagnosis of untreated or unstable behavioural or neurodevelopmental disorders (e.g., ADHD, autism spectrum disorders).

Exclusion criteria included: incomplete or missing clinical documentation; prior or concurrent treatment with other medications that affect bladder function (e.g., tricyclic antidepressants, beta-agonists); history of urological surgery or invasive bladder procedures during the study period.

### 2.2. Treatment

Patients were retrospectively categorized into two groups based on the treatment documented in their medical records. Oxybutynin was prescribed at 0.5 mg/kg/day, whereas LEVIGON™ PRO was administered according to the fixed regimen used in routine clinical practice (2 sachets/day) across the included age range.

− Group A (Combination Therapy): treated with LEVIGON™ PRO (Sanitpharma, Milan, Italy), 2 sachets/day, orally on an empty stomach, alongside oxybutynin (0.5 mg/kg/day). LEVIGON™ PRO is a patented formulation containing, per sachet, 2.5 g of myo-inositol, 56.25 mg of microlipodispersed magnesium, 200 mcg of folic acid, and 20 mg of vitamin C. Throughout the treatment period, patients were instructed to take the supplement at approximately the same time each day, in the fasted state.
− Group B (Standard Therapy): treated with oxybutynin (0.5 mg/kg/day).

Treatment duration in both groups was 4 months, according to the standardized hospital protocol. Follow-up visits at approximately 4 and 16 weeks were part of routine clinical practice, and data were retrospectively extracted when available.

### 2.3. Pharmacodynamic Rationale for Adjunctive LEVIGON™ PRO Therapy

**Figure 2:**
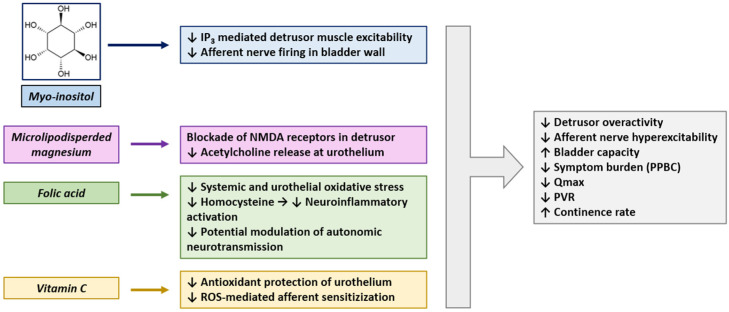
Mechanistic framework of nutraceutical modulation in paediatric overactive bladder (OAB).

The rationale for using LEVIGON™ PRO as adjunctive therapy in paediatric OAB is grounded in the complementary pharmacodynamic actions of its nutraceutical components. Each ingredient targets specific neuro-urological or metabolic mechanisms that contribute to detrusor overactivity, bladder hypersensitivity, and impaired continence control (Figure 2).

Myo-inositol, a polyol precursor of inositol phosphates and phosphatidylinositol, has shown neuro-modulatory and insulin-sensitizing effects in both clinical and preclinical models [22]. It contributes to neuronal signal transduction pathways implicated in detrusor overactivity and bladder afferent sensitization, particularly via regulation of phosphoinositide 3-kinase (PI3K) and Akt signalling cascades in bladder smooth muscle and urothelium [18]. Myo-inositol is a key component of phosphoinositide-mediated signaling pathways implicated in neuronal plasticity. Although direct evidence in the bladder is lacking, it is plausible that such mechanisms might modulate cholinergic transmission and neuroplasticity relevant to urgency symptoms [23]. In adults with PCOS, myo-inositol supplementation has been shown to improve metabolic and hormonal profiles, with enhanced insulin sensitivity and reduced hyperandrogenism, which may indirectly contribute to improvements in bladder function and urgency symptoms, although direct evidence in OAB populations is currently lacking [24].

Magnesium, particularly in bioaccessible formulations such as microlipodispersed forms [25], may exert smooth muscle relaxation effects by acting as a physiological calcium antagonist, thereby attenuating detrusor overactivity [26]. Although direct pediatric trials are lacking, magnesium plays a key role in neuromuscular excitability, and preliminary clinical and epidemiological evidence suggests that magnesium status may influence lower urinary tract symptoms, including overactive bladder and nocturnal enuresis [27]. Moreover, magnesium may block NMDA receptors expressed in both detrusor muscle and the urothelium, thereby reducing glutamatergic excitatory signaling involved in bladder hypersensitivity [28]. The enhanced absorption offered by microlipodispersed formulations may be particularly relevant in paediatric populations, where intestinal absorption kinetics can be variable [29].

Folic acid, though classically associated with methylation cycles and neural development, also plays a role in maintaining autonomic nervous system balance [30]. Recent data suggest that folate deficiency may exacerbate detrusor instability via oxidative stress and impaired nitric oxide signalling [31]. Furthermore, folate interacts with homocysteine metabolism, which has been implicated in vascular dysfunction and altered urothelial perfusion, a potential contributing factor in OAB pathogenesis [32]. This vascular component may compromise bladder wall oxygenation and compliance, aggravating detrusor overactivity [33].

Vitamin C serves as a potent antioxidant which may have the ability to mitigate oxidative stress-mediated bladder hypersensitivity [34]. Preclinical studies have highlighted the role of oxidative stress and afferent nerve hyperexcitability in urothelial dysfunction and bladder overactivity, suggesting that antioxidant strategies, including vitamin C, may provide uroprotective benefits by preserving urothelial barrier integrity and modulating afferent signaling [35]. Its additional role in supporting collagen synthesis and extracellular matrix integrity may contribute to improved bladder compliance. Moreover, its role in enhancing endothelial nitric oxide synthase (eNOS) activity may indirectly improve bladder perfusion and compliance [36].

This Figure 2 illustrates the hypothesized synergistic mechanisms through which the components of the nutraceutical formulation LEVIGON™ PRO (myo-inositol, microlipodispersed magnesium, folic acid, and vitamin C) may target pathophysiological pathways implicated in paediatric OAB. These mechanisms include neuromodulation, oxidative stress reduction, detrusor smooth muscle relaxation, and improved urothelial signalling and perfusion. Collectively, they may contribute to improved bladder capacity, reduced detrusor overactivity, and increased continence rates.

### 2.4. Assessment of Efficacy

Efficacy was assessed through structured retrospective analysis of clinical documentation and caregiver-reported outcome measures approximately at three time points: baseline (T0), day 28 (T1), and day 112 (T2). Given the retrospective design, completeness of diary-based and caregiver-reported measures varied across timepoints according to the availability of documentation in the medical records. All clinical assessments were performed by trained paediatric urology personnel using standardized tools.

The primary efficacy endpoint was the proportion of patients achieving complete daytime urinary continence by T2, defined as seven consecutive days without any wetting episodes, as documented in caregiver diaries and validated by physician review. This binary outcome was chosen based on its clinical relevance and its adoption in similar paediatric overactive bladder (OAB) trials [4,37].

Secondary outcomes included the following clinical parameters:

Weekly incontinence episodes, measured as the mean number of involuntary wetting events per week, documented using structured bladder diaries compiled by caregivers, and corroborated through outpatient clinical notes [1].

Daily voiding frequency, calculated as the average number of voluntary micturitions per 24 h, based on three-day diary entries prior to each visit [38].

Bladder wall thickness (BWT), assessed via suprapubic ultrasonography using a standardized bladder-filling protocol (minimum 50% of estimated bladder capacity), at T0 and T2. Measurements were obtained in transverse view at the anterior bladder wall, following current paediatric urology imaging guidelines [39].

Uroflowmetry parameters, including Maximum flow rate (Qmax, mL/s), Mean voided volume (mL), Post-void residual volume (PVR, mL). These parameters were measured using non-invasive uroflowmetry followed by portable bladder ultrasound (bladder scan) within 5 min post-voiding, conducted at T0 and T2. Only bell-shaped uroflowmetry curves considered clinically interpretable and obtained with a voided volume deemed adequate in routine paediatric clinical practice were included in the analysis. Because of the retrospective design, duplicate consecutive uroflowmetry measurements were not systematically available for all patients [40].

Symptom burden quantified using the Patient Perception of Bladder Condition (PPBC), a validated single-item Likert scale ranging from 1 (“no problems at all”) to 5 (“many severe problems”), previously validated in paediatric voiding dysfunction studies [41].

Additionally, a post hoc exploratory metabolic analysis was performed in a subgroup of 34 children with a previously documented diagnosis of impaired fasting glucose (IFG). In these patients, the following metabolic indices were collected at T0, T1, and T2: Fasting plasma glucose (mg/dL), Fasting insulin (μU/mL), Homeostatic Model Assessment of Insulin Resistance (HOMA-IR), calculated as HOMA-IR = [fasting insulin (μU/mL) × fasting glucose (mg/dL)]/405.

This sub-analysis aimed to investigate potential associations between metabolic dysfunction and treatment response, in light of emerging evidence linking insulin resistance and OAB pathophysiology [14,15].

### 2.5. Assessment of Safety and Tolerability

Treatment safety and tolerability were monitored throughout the study duration. Adverse events (AEs) were identified via systematic review of clinical progress notes and spontaneous caregiver reporting at T1 and T2. All AEs were classified by severity and assessed for causal relationship with the treatment, in accordance with WHO-UMC criteria.

Treatment adherence was assessed retrospectively through structured review of clinical documentation and caregiver-reported adherence as recorded in medical records. Notes from routine follow-up visits and telephone contacts were examined to evaluate the degree of compliance with the prescribed supplementation regimen. When available, information regarding the estimated number of missed doses or interruptions in treatment was extracted from caregiver narratives or physician annotations. Adherence was qualitatively categorized as “high” (≥80% of doses taken), “moderate” (50–79%), or “low” (<50%) in accordance with accepted thresholds in paediatric nutraceutical studies [42].

### 2.6. Statistical Analysis

Statistical analyses were performed using GraphPad Prism (version 8.0.2) software. Continuous variables are presented as the mean ± standard deviation (SD), while categorical variables are expressed as counts and percentages. Baseline demographic and clinical characteristics were compared between groups using unpaired Student’s *t*-tests or Mann–Whitney U tests, as appropriate, based on data distribution. Categorical variables were compared using the χ^2^ test or Fisher’s exact test when applicable.

The primary endpoint, defined as the proportion of patients achieving complete daytime urinary continence at Day 112, was analysed using the χ^2^ test. Absolute risk difference and number needed to treat (NNT) were calculated descriptively.

For longitudinal continuous outcomes (incontinence episodes, voiding frequency, mean voided volume, post-void residual, PPBC score, bladder wall thickness, and Qmax), changes over time between treatment groups were analysed using linear mixed-effects models, with treatment group, time, and their interaction included as fixed effects, and patient identifier as a random effect. This approach accounts for within-subject correlations and unbalanced repeated measures inherent to retrospective data. No formal imputation of missing repeated-measures data was performed; longitudinal analyses were based on available observations extracted from the medical records.

A post hoc exploratory subgroup analysis was conducted in patients with impaired fasting glucose (IFG). Longitudinal changes in metabolic parameters (fasting glucose, fasting insulin, and HOMA-IR) were analysed using the same mixed-effects modelling approach.

All statistical tests were two-tailed, and a *p* value < 0.05 was considered statistically significant. Given the exploratory nature of secondary and subgroup analyses, no formal adjustment for multiple comparisons was applied.

## 3. Results

A total of 120 paediatric patients (age 5–15 years) with a confirmed diagnosis of OAB met eligibility criteria and were included in the analysis (Table 1). Patients were retrospectively assigned to treatment cohorts based on therapy documented in the medical record: Group A (Combination Therapy) received oxybutynin plus LEVIGON™ PRO (myo-inositol, microlipodispersed magnesium, folic acid, vitamin C), whereas Group B (Standard Therapy) received oxybutynin alone.

Baseline demographic and anthropometric variables (age, sex distribution, weight, height, BMI) were comparable between groups (Table 1). No statistically meaningful between-group differences were identified at baseline in symptom burden, incontinence frequency, voiding frequency, or urodynamic measures (Table 2).

An exploratory analysis was conducted in a subset of 34 participants with a documented history of impaired fasting glucose (IFG), to explore potential associations with clinical outcomes (Table 3).

By Day 112 (T2), 37 out of 60 patients (61.7%) in Group A achieved complete daytime urinary continence, compared with 29 out of 60 patients (48.3%) in Group B (Table 4). This corresponds to an absolute risk difference of 13.4%, yielding a number needed to treat (NNT) of approximately 7.5 to achieve one additional continence responder with adjunctive nutraceutical therapy. The between-group difference did not reach statistical significance (χ^2^ test, *p* = 0.14), although a numerically higher response rate was observed in the combination group. Interim analysis at Day 28 (T1) showed a numerically higher proportion of responders in Group A (31.7%) compared to Group B (23.3%), but the difference was not statistically significant (χ^2^ test, *p* = 0.31).

**Table 4:** Proportion of children achieving complete daytime urinary continence at T1 and T2.

Timepoint	Group A (Combination Therapy; N = 60)	Group B (Oxybutynin; N = 60)	*p*-Value
T0 (Baseline)	0 (0%)	0 (0%)	-
T1 (Day 28)	19 (31.7%)	14 (23.3%)	0.31
T2 (Day 112)	37 (61.7%)	29 (48.3%)	0.14

Across most prespecified secondary endpoints, mean changes from baseline to Day 112 numerically favoured the combination therapy group (Table 2). Improvements were observed in both treatment arms, and several between-group differences reached nominal statistical significance over time; however, these findings should be interpreted cautiously given the retrospective non-randomized design, and the exploratory nature of secondary analyses.

At the interim assessment (Day 28, T1), both groups showed early improvements across most endpoints; however, between-group differences were not statistically significant at this time point, suggesting a progressive treatment effect that became more evident with longer treatment duration.

Weekly incontinence episodes declined markedly in both groups. In Group A, episodes decreased from 11.2 ± 3.1 to 3.1 ± 1.4 per week, corresponding to a relative reduction of approximately 72%, whereas Group B showed a reduction from 10.9 ± 3.2 to 4.5 ± 1.6 (−59%). The between-group difference in change over time was statistically significant (group × time interaction, *p* < 0.05), indicating a more pronounced clinical response with combination therapy.

Voiding frequency over 24 h improved in both groups, with a significantly greater reduction in Group A (from 7.8 ± 1.4 to 5.7 ± 1.0 voids/day; −27%) compared with Group B (from 7.6 ± 1.4 to 6.2 ± 1.1; −18%) (*p* < 0.05).

Mean voided volume increased by 32 mL in Group A (from 120.5 ± 39.1 to 152.5 ± 44.1 mL; +27%) and by 24 mL in Group B (from 118.7 ± 37.5 to 143.1 ± 41.2 mL; +20%), with a statistically significant between-group difference in longitudinal change (*p* < 0.05). Similarly, post-void residual volume declined more markedly in Group A (−43%) than in Group B (−33%) (*p* < 0.05), suggesting improved bladder emptying with adjunctive therapy.

PPBC scores improved significantly in both arms; however, the reduction was significantly greater in Group A (−1.6 points; −41%) compared with Group B (−1.0 point; −26%) (*p* < 0.05), reflecting a superior patient-perceived benefit.

Regarding anatomical and urodynamic parameters assessed at baseline and Day 112, bladder wall thickness decreased more prominently in the combination group (−21% vs. −15%), and maximum flow rate (Qmax) increased to a greater extent (+31% vs. +25%), with both endpoints showing significant between-group differences over time (*p* < 0.05).

Overall, the secondary outcome analyses numerically favoured the combination group, but these findings should be interpreted with caution given the observational nature of the study.

Thirty-four patients had documented impaired fasting glucose (IFG) at baseline (Table 3). By Day 112, fasting glucose, fasting insulin and HOMA-IR were significantly lower in the combination group compared with oxybutynin alone (*p* = 0.008, *p* = 0.002 and *p* < 0.001, respectively). At Day 28, fasting insulin and HOMA-IR were already significantly reduced in the combination group (*p* = 0.040 and *p* = 0.024), while fasting glucose showed a non-significant trend (*p* = 0.080) (Table 5). Because this subgroup was small and the analysis was exploratory, these findings should be considered hypothesis generating.

No serious adverse events were documented in either group. The most commonly recorded treatment-emergent events were dry mouth (6.7% in Group A vs. 8.3% in Group B), constipation (5.0% vs. 6.7%), and mild abdominal discomfort (3.3% in both groups). No discontinuations were attributed to adverse events. However, because adverse-event ascertainment relied on retrospective review of clinical progress notes and spontaneous caregiver reporting, mild or transient symptoms may have been under-recorded. Documentation from follow-up visits and caregiver communications suggested generally good adherence in both groups; however, adherence was assessed retrospectively and qualitatively, and objective sachet counts were inconsistently available. Therefore, adherence could not be formally compared between groups.

## 4. Discussion

This retrospective observational study suggests that adjunctive nutraceutical therapy with a formulation containing myo-inositol, microlipodispersed magnesium, folic acid, and vitamin C, when combined with oxybutynin, may provide additional clinical benefit in paediatric OAB compared with oxybutynin alone. Although both groups improved over time, consistent with the known efficacy of antimuscarinic therapy, the combination arm exhibited a more favourable trajectory across prespecified secondary endpoints, including symptom frequency, patient-perceived burden, and objective functional/anatomical measures.

With respect to the primary outcome, complete daytime continence at Day 112 was achieved in 61.7% of children receiving combination therapy versus 48.3% in those treated with oxybutynin alone (absolute risk difference 13.4%; NNT ≈ 7.5). While this between-group difference did not reach statistical significance (*p* = 0.14), the magnitude of effect may still be clinically meaningful in a condition that substantially impairs daily functioning, self-esteem, and family burden, particularly when therapeutic options are limited and long-term tolerability can constrain adherence. Importantly, improvements in several secondary outcomes were significantly greater in the combination group, supporting the hypothesis of an additive benefit beyond antimuscarinic therapy alone.

Notably, the treatment effect appeared progressive. At the interim assessment (Day 28), both groups showed early improvements, whereas between-group differences were not statistically significant at that time point. By Day 112, the combination arm demonstrated significantly greater longitudinal improvements (group × time interaction, *p* < 0.05) in weekly incontinence episodes, voiding frequency, mean voided volume, post-void residual volume, and PPBC score. The greater reduction in PPBC further supports the real-world relevance of the observed changes, as patient-perceived symptom burden is highly consequential in paediatric voiding dysfunction and often drives treatment persistence.

The observed functional and anatomical improvements are biologically plausible in light of the proposed pharmacodynamic rationale of the tested nutraceutical formulation [26,43]. Because the adjunctive intervention evaluated in the present study consisted of a multi-component formulation, the relative contribution of each individual ingredient cannot be isolated from the current dataset. Accordingly, conclusions should be referred to the formulation as a whole rather than to single ingredients. Myo-inositol and magnesium may plausibly have contributed to the observed effects; however, folic acid and vitamin C were present at doses close to nutritional reference values rather than at pharmacological levels and were therefore less likely to represent the principal drivers of the clinical response. Nevertheless, a contributory role of these components cannot be completely excluded. Myo-inositol may influence insulin signalling and neuronal pathways involved in autonomic regulation, while magnesium can modulate smooth muscle excitability and may attenuate detrusor overactivity via calcium-antagonist effects. The potential contribution of vitamin C and folate-related pathways is more difficult to define in the present context, particularly given their nutritional-support rather than pharmacological dosing. Although mechanistic inference remains indirect in the absence of biomarker assessment, these considerations provide a biologically plausible interpretative framework for the observed clinical findings.

An additional, hypothesis-generating finding emerged from the exploratory metabolic analysis in children with IFG. In this subgroup, by Day 112 fasting glucose, fasting insulin, and HOMA-IR were significantly lower in the combination group than in the oxybutynin-only group (*p* = 0.008, *p* = 0.002, and *p* < 0.001, respectively), with insulin and HOMA-IR differences already evident at Day 28 (*p* = 0.040 and *p* = 0.024). This aligns with prior evidence linking insulin resistance to OAB symptoms via mechanisms such as sympathetic overactivation, increased detrusor sensitivity, and low-grade inflammation [14,15]. While causality cannot be inferred and the subgroup sample size is limited, these findings raise the possibility that metabolic modulation may contribute to symptom improvement in metabolically vulnerable children and may help identify responders for future stratified trials. Accordingly, the metabolic findings observed in the present study should be interpreted as exploratory and hypothesis generating, rather than as evidence of an established causal role of IR in paediatric OAB. More broadly, improvements in metabolic indices may have contributed to clinical benefit through modulation of autonomic tone and endothelial function and, potentially, through attenuation of low-grade systemic inflammation. Experimental data suggest that insulin resistance–driven inflammation may disrupt urothelial barrier function, promote mast cell activation, and amplify ATP-mediated afferent signalling, thereby facilitating urgency and frequency symptoms [44]. Accordingly, nutraceutical components such as myo-inositol, magnesium, and vitamin C may exert pleiotropic effects extending beyond glycaemic control. Nevertheless, these mechanistic interpretations remain speculative and should be confirmed in prospective studies incorporating biomarker profiling.

While the FOXY trial [7] and other paediatric studies have established the short-term efficacy of antimuscarinics such as oxybutynin and solifenacin, tolerability concerns and treatment-emergent adverse events may limit long-term persistence. In the present cohort, dry mouth and constipation were the most common TEAEs, no serious adverse events occurred, and tolerability appeared comparable between the two regimens.

The concept of adjunctive nutraceutical strategies in OAB is supported by emerging mechanistic and clinical evidence. Magnesium has been shown to modulate detrusor overactivity through calcium antagonism and smooth muscle relaxation, with clinical data suggesting a potential role in reducing bladder discomfort and urgency [26]. In parallel, myo-inositol may improve insulin sensitivity and neuromodulatory signaling pathways implicated in bladder function [18]. Given the recognized association between metabolic dysfunction and OAB [14,15], the combination of these agents represents a biologically plausible adjunctive approach. However, paediatric evidence remains limited, and the present study contributes real-world data in a relatively large clinical cohort.

Important limitations must be acknowledged. The retrospective and non-randomised design inherently limits causal inference and introduces potential sources of bias. Uroflowmetry measurements were retrospectively analysed as available in routine clinical practice, and duplicate consecutive traces were not systematically available for all patients. Because of the retrospective design, treatment allocation was not protocol-driven or randomized, but reflected routine clinical prescribing as documented in the medical records. The reasons underlying adjunctive LEVIGON™ PRO prescription were not prospectively standardized, which may have introduced selection bias and confounding by indication. Although baseline demographic and clinical characteristics were comparable between groups, residual confounding by unmeasured variables cannot be excluded. In addition, bowel dysfunction and constipation management were not systematically recorded in a standardized format across all charts and therefore could not be formally compared between groups. Given the recognized interaction between constipation and paediatric OAB, residual confounding related to bowel status cannot be excluded. The adjunctive nutraceutical was administered as a fixed regimen and was not titrated according to age or body weight, which may have introduced heterogeneity in exposure across the included age range. In addition, adverse-event ascertainment relied on retrospective review of clinical progress notes and spontaneous caregiver reporting, which may have underestimated mild or transient symptoms. Moreover, treatment adherence was reconstructed retrospectively from clinical documentation and caregiver reports; therefore, objective measures such as systematic sachet counts were not consistently available, and differential family engagement, reporting behaviour, or clinician follow-up intensity cannot be excluded as potential contributors to the observed between-group differences.

Nevertheless, the study included a relatively large and well-characterised paediatric cohort managed in a real-world clinical setting. Clinical outcomes were routinely and systematically recorded during standard follow-up visits, and the integration of subjective symptom scores with objective functional and anatomical measures (uroflowmetry and ultrasound) strengthens the overall robustness of the findings. Finally, the exploratory metabolic sub-analysis provides a plausible mechanistic link between insulin resistance, low-grade inflammation, and OAB in children, warranting further investigation in prospective studies

These findings open several avenues for future research. First, a randomised, double-blind, placebo-controlled trial is needed to validate the observed benefits and clarify causality. Second, mechanistic studies incorporating biomarkers of neuroinflammation, oxidative stress, and endothelial function could elucidate the pathways through which these nutraceuticals exert their effects. Third, stratification based on metabolic profiles (e.g., HOMA-IR, BMI, adipokines) may identify responders to tailored adjunctive therapy. Lastly, longer follow-up would clarify the durability of response, recurrence rates, and whether combination therapy facilitates earlier withdrawal or dose reduction of antimuscarinics.

Taken together, this data support further investigation of adjunctive nutraceutical strategies in paediatric OAB, particularly in metabolically vulnerable children, in prospective controlled studies designed to clarify efficacy, safety, and potential responder profiles.

## 5. Conclusions

In this retrospective observational study, adjunctive nutraceutical therapy combined with oxybutynin was associated with greater improvements in several secondary clinical outcomes in children with overactive bladder, with a favourable tolerability profile. Although complete daytime continence at Day 112 did not differ significantly between groups, the overall pattern of findings suggests a potential additive benefit. Exploratory results indicate that metabolic factors, including insulin resistance, may influence treatment response. Prospective randomised controlled trials are needed to confirm these findings and clarify causality.

## Figures and Tables

**Table 1:** Baseline demographic and anthropometric characteristics.

Parameter	Group A (Combination Therapy; N = 60)	Group B (Oxybutynin; N = 60)	*p*-Value
Male, n (%)	37 (61.7)	34 (56.7)	0.58
Age (years ± SD)	8.9 ± 1.7	9.1 ± 1.6	0.51
Weight (kg ± SD)	32.9 ± 8.1	33.6 ± 7.5	0.62
Height (m ± SD)	1.32 ± 0.11	1.34 ± 0.10	0.30
BMI (kg/m^2^ ± SD)	18.5 ± 2.3	18.6 ± 2.1	0.80

**Table 2:** Secondary clinical endpoints at baseline (T0), Day 28 (T1), and Day 112 (T2).

Parameter	T0—Group A (Combination Therapy; N = 60)	T0—Group B (Oxybutynin; N = 60)	T1—Group A (Combination Therapy; N = 60)	T1—Group B (Oxybutynin; N = 60)	T2—Group A (Combination Therapy; N = 60)	T2—Group B (Oxybutynin; N = 60)
Incontinence episodes/week	11.2 ± 3.1	10.9 ± 3.2	6.2 ± 2.4	6.7 ± 2.6	3.1 ± 1.4 *	4.5 ± 1.6
Voiding frequency/24 h	7.8 ± 1.4	7.6 ± 1.4	6.5 ± 1.3	6.8 ± 1.2	5.7 ± 1.0 *	6.2 ± 1.1
Mean voided volume (mL)	120.5 ± 39.1	118.7 ± 37.5	137.0 ± 40.5	132.0 ± 38.7	152.5 ± 44.1 *	143.1 ± 41.2
Post-void residual (mL)	16.3 ± 10.1	16.5 ± 10.5	11.9 ± 8.1	13.5 ± 7.8	9.3 ± 6.4 *	11.0 ± 7.0
PPBC score (1–5)	3.9 ± 0.8	3.8 ± 0.9	3.0 ± 0.7	3.3 ± 0.8	2.3 ± 0.7 *	2.8 ± 0.8
Bladder wall thickness (mm)	5.3 ± 0.7	5.3 ± 0.8	—	—	4.2 ± 0.6 *	4.5 ± 0.7
Qmax (mL/s)	10.3 ± 3.4	10.2 ± 3.5	—	—	13.5 ± 3.7 *	12.7 ± 3.9

Data are presented as the mean ± SD. * *p* < 0.05 for between-group differences over time, derived from linear mixed-effects models including treatment group, time, and group × time interaction as fixed effects, and patient as a random effect. Bladder wall thickness and Qmax were assessed only at baseline (T0) and Day 112 (T2).

**Table 3:** Baseline characteristics of patients with impaired fasting glucose (IFG).

Parameter	Group A (Combination Therapy; N = 16)	Group B (Oxybutynin; N = 18)	*p*-Value
Weight (kg ± SD)	35.7 ± 6.5	36.9 ± 6.8	0.60
Height (m ± SD)	1.31 ± 0.09	1.33 ± 0.08	0.49
BMI (kg/m^2^ ± SD)	20.5 ± 2.0	20.8 ± 1.8	0.64
Fasting plasma glucose (mg/dL ± SD)	108.2 ± 6.1	109.4 ± 5.2	0.55
Fasting insulin (µU/mL ± SD)	21.2 ± 5.7	22.6 ± 6.3	0.49
HOMA-IR (±SD)	5.5 ± 1.8	5.9 ± 1.9	0.54

**Table 5:** Changes in metabolic parameters over time in IFG patients treated with combination therapy or oxybutynin alone.

Parameter	Group A (Myo + Oxy)—T0 N = 16	Group A—T1	Group A—T2	Group B (Oxy Only)—T0 N = 18	Group B—T1	Group B—T2
Fasting glucose (mg/dL)	108.2 ± 11.0	101.5 ± 8.5	96.8 ± 6.9 **	109.4 ± 10.5	107.3 ± 10.2	104.9 ± 9.7
Fasting insulin (μU/mL)	21.2 ± 4.8	16.5 ± 4.3 *	12.1 ± 4.0 **	22.6 ± 4.9	19.8 ± 4.7	17.2 ± 4.8
HOMA-IR	5.6 ± 1.6	4.1 ± 1.3 *	2.9 ± 1.0 ***	6.1 ± 1.7	5.2 ± 1.4	4.4 ± 1.3

Data are presented as the mean ± SD.* *p* < 0.05, ** *p* < 0.01, *** *p* < 0.001 for between-group comparisons (Group A vs. Group B) at the corresponding timepoint.

## Data Availability

The data presented in this study are available on request from the corresponding author. The data are not publicly available due to privacy and ethical restrictions, as the study was based on retrospectively collected anonymised paediatric clinical data from medical records. Data sharing is subject to compliance with applicable institutional policies and EU Regulation 2016/679 (GDPR), and must not compromise patient confidentiality.
